# Determinants of Residents’ Approach–Avoidance Responses to the Personal Carbon Trading Scheme: An Empirical Analysis of Urban Residents in Eastern China

**DOI:** 10.3390/ijerph18020822

**Published:** 2021-01-19

**Authors:** Daoyan Guo, Hong Chen, Ruyin Long, Shaohui Zou

**Affiliations:** 1 Centre for Energy Economics and Management Research, School of Management, Xi’an University of Science and Technology, Xi’an 710054, China; guodaoyan@xust.edu.cn; 2 School of Economics and Management, China University of Mining and Technology, Xuzhou 221116, China; longruyin@cumt.edu.cn

**Keywords:** behavioral willingness, downstream carbon emissions, empirical analysis, government policy, personal carbon trading scheme

## Abstract

The personal carbon trading (PCT) scheme is a policy instrument for reducing downstream carbon emissions and creating a green lifestyle, and a bottleneck hampering its implementation is the behavioral willingness of residents. Due to a pre-existing stimulus-response association, the willingness of residents can be intuitively reflected by their approach–avoidance responses. This study theoretically analyzed the mechanisms for influencing residents’ approach–avoidance responses towards the personal carbon trading scheme based on open-ended interviews, and empirically examined the mechanisms by developing rating scales and conducting questionnaire surveys on urban residents in eastern China. Findings showed that residents’ approach–avoidance responses could be negatively affected by their preference for comfort, whereas they were positively impacted by their ecological values, sense of social responsibility, cognition of the behaviors for carbon emission reduction, and cognition of personal carbon trading. In terms of subjective norms, the culture of environmentalism had a positive effect on residents’ responses to PCT scheme, while the culture of consumerism caused a negative impact on their responses. Furthermore, the perceived behavioral controls of residents partially mediated the relationships between their psychological characteristics and approach–avoidance responses. Finally, primary and pivotal suggestions were proposed for nudging Chinese urban residents towards approaching the personal carbon trading scheme, which provide theoretical support and practical guidance for its implementation.

## 1. Introduction

The environmental risks and health problems caused by global climate change are of great concern to the whole of society. Climatic disasters, such as forest fires, hurricanes, droughts, and heat waves pose serious threats to human infrastructure, safety, and health [1,2]. The Intergovernmental Panel on Climate Change stated that increased carbon dioxide emissions are the largest contributor to global climate change, and these are extremely likely (at least 95% certainty) to be caused by human activities. With the growth of the global economy and the improvement of living standards, the amount and percentage of carbon emissions caused by household energy consumption show increasing trends [3,4], and downstream carbon emissions figure prominently in national carbon emissions [5]. For example, Tian et al. (2014) found that 35% of China’s carbon footprint was attributable to household consumption [6]. Su et al. (2017) pointed out that about a quarter of the carbon dioxide emissions were attributable to the activities of residents in Singapore [7]. Therefore, residents should play an important role in the abatement of carbon emissions. It is urgent to cut downstream carbon emissions.

In consideration of the negative externality and long-term effect of carbon emissions, it is necessary to rely on government policies to reduce downstream carbon emissions. A personal carbon trading (PCT) scheme, which is regarded as a market-based tool for emissions reduction, is deemed to offer guidance to residents for carbon emission reduction with carbon price signals. In 17 April 2017, Guangdong Provincial Development and Reform Commission released the Interim Regulation for the Emission Reductions Certified by the Carbon Generalized System of Preferences (ERCC). This regulation marked the beginning of the incorporation of ERCC with the complementary mechanisms of a carbon trading market. Hence, related enterprises and individuals in the pilot areas of the Carbon Generalized System of Preferences can formally participate in carbon trading by voluntarily reducing carbon emissions (e.g., by travelling by bus and saving water and electricity) and increasing green carbon sinks. There is little doubt that a rudimentary PCT market has come into being in China, and the market is bound to be broadened in the near future.

The PCT scheme is generally characterized by efficiency and social equity [8], and there are no substantive technical obstacles for its implementation [9]. By means of the PCT market, it is highly likely that residents will take the initiative in reducing carbon emissions and thereby contribute to the promotion of low carbon behaviors, lifestyles, and consumption patterns [10]. Despite the various advantages of the PCT scheme, the feasibility of its implementation largely depends on the behavioral willingness of its participants; that is, residents [11]. The evidence shows that residents’ behavioral willingness to accept and participate in the scheme could be influenced by individual factors; these include the cognition of climate change [12], perception of environmental threat [13], expected behaviors for energy conservation and emissions reduction [13], and environmental awareness [14]. The scheme itself also affects residents’ attitudes, such as the pattern for implementing the scheme [5], the method for allocating the initial carbon allowance [15], and the household activities included in the measurement of the carbon emissions [16]. In addition, the behavioral willingness of residents can be intuitively reflected by their approach–avoidance responses (AAR), due to a pre-existing stimulus-response association [17]. In fact, all behaviors are related to approach or avoidance [18]. Approaching and avoiding the PCT scheme indicates that residents accept and oppose the scheme, respectively; thus, their AAR towards the PCT scheme is an important evaluation criterion of its successful implementation.

A few researchers tried to evaluate its feasibility by investigating the level of public support, but there is no reported literature on the residents’ AAR to the PCT scheme and the associated influencing factors. Taking into account the fact that individual behavioral choices are not entirely rational, the theory of planned behavior, value–attitude–behavior theory, value–belief–norm theory, and the theory of responsible environmental behavior can be adopted to analyze the determinants of residents’ AAR. The theory of planned behavior is a mainstream theory for describing behavior and behavioral intentions, which can together be shaped by attitudes, subjective norms, and perceived behavioral controls [19]. The value–attitude–behavior theory stresses the importance of individual value in taking environmental actions, and the value influences behavior indirectly through attitude [20]. The value–belief–norm theory states that an individual’s decision on pro-environmental behaviors is driven by value, belief, and personal norms in sequence [21]. The theory of responsible environmental behavior proposed by Hines, Hungerford, and Tomera (1987) asserted that the following factors have considerable impacts on the intention of adopting responsible environmental behavior: knowledge of issues, knowledge of action strategies, locus of control, attitudes, verbal commitment, and sense of responsibility [22]. Locus of control refers to the degree to which people believe that they have control over their lives. Sia et al. (1986) verified that the following seven factors are significantly influential in motivating individuals to take responsible environmental actions: the level of environmental sensitivity, perceived knowledge of environmental action strategies, perceived skill in using environmental action strategies, psychological sex role classification, individual locus of control, group locus of control, and attitude towards pollution [23]. The above theories provided theoretical basis for exploring the determinants of residents’ AAR. The purpose of this study was to systematically clarify the mechanism for influencing residents’ AAR towards the PCT scheme and to thereby provide policy suggestions for nudging Chinese urban residents towards approaching the scheme. This study provides a breakthrough in the research of low-carbon development theory, because it takes a novel perspective—approach–avoidance—to investigate residents’ responses to the PCT scheme. The residents’ responses are crucial for the selection of mandatory or voluntary modes and the associated policy developments. The construction of the theoretical framework for influencing residents’ AAR towards the PCT scheme is a key contribution to the theories of environmental behaviors, because it extends the existing knowledge within the limits of the behavioral willingness towards the scheme. The primary and pivotal suggestions are conducive to the guidance of Chinese urban residents in approaching the PCT scheme, which will thereby contribute to its implementation.

## 2. Materials and Methods

### 2.1. Definition of Influencing Factors

The existing theories such as planned behavior theory, value–attitude–behavior theory, value–belief–norm theory, and theory of responsible environmental behavior have explained the factors influencing individual environmental behaviors from different perspectives. As a systematic methodology that involves the conceptualization of data and the construction of hypotheses, grounded theory was adopted to qualitatively explore the influencing factors of residents’ responses. The procedure was mainly implemented as follows. Firstly, obtain first-hand information from in-depth interviews of representative urban residents in eastern China. Secondly, make open, axial, and selective coding analyses of the original statements. Thirdly, test the theoretical saturation to determine the influencing factors of residents’ AAR to the scheme. In consideration of the forward-looking nature of the PCT scheme, interviewees were required to be aware of climate change and have a basic understanding of PCT. Moreover, stratified sampling was applied to select representative residents in order to make the demographic distributions of respondents reasonable and to be consistent with reality. The interview questions were centered on the PCT scheme, such as “Are you willing to accept the PCT scheme?” and “Why do you accept (reject) the scheme?” Based on the above analyses, three kinds of factors were summarized, and they are shown in Table 1. The psychological characteristics include ecological value, sense of social responsibility, preference for comfort, cognition of the behaviors for carbon emission reduction, and cognition of PCT. The subjective norms are represented by the culture of consumerism and the culture of environmentalism.

### 2.2. Construction of Research Hypothesis

The value–attitude–behavior theory stresses the importance of individual value in taking environmental actions, and that value influences behavior indirectly through attitude [20]. The value–belief–norm theory states that an individual’s decision on pro-environmental behaviors could be driven by value (e.g., egoistic value, altruistic value, ecological value) [21]. The theory of responsible environmental behavior proposed by Hines, Hungerford, and Tomera (1987) also pointed out that individual value has considerable impacts on the intention of adopting responsible environmental behavior [22]. The ecological value is supposed to be a factor influencing residents’ AAR towards the PCT scheme. Ecological value is how people perceive the importance of improving environmental quality. Researchers found that environmental value had positive influences on ecological intentions and pro-environmental behaviors [21,24,25]. For example, an investigation based on 275 undergraduate students indicated that the students’ intentions of adopting pro-environmental behavior were significantly affected by the sustainability value in the context of global climate change [26]. Therefore, residents with high ecological values are highly likely to perform environmental behaviors and accept a PCT scheme. Based on the above analyses, the following hypothesis was proposed.

**Hypothesis 1** **(H1).** *Residents’ ecological values can positively influence their AAR towards the PCT scheme.*

Based on the theory of responsible environmental behavior, the sense of responsibility was an important predictor of individual intentions towards future involvement in environmental actions [22,25,27]. The sense of responsibility indicates an awareness of having an obligation to make contributions to the society. The evidence showed that an individual would behave environmentally when they had a high sense of social responsibility, which was consistent with the theory of responsible environmental behavior. For instance, Hines, Hungerford, and Tomera (1987) found that the higher the sense of responsibility was for alleviating environmental problems and improving environmental quality, the greater the likelihood would be of adopting environmental behaviors [22]. Yang, Zhang, and Zhao (2016) pointed out that people with a high sense of environmental responsibility were more likely to take both direct and indirect energy curtailment actions [28]. A survey of Chinese consumers also revealed that environmental responsibility could promote environmental concerns and enhance green consumption [29]. Thus, we assume that there are positive influences caused by residents’ sense of social responsibility on their AAR towards the PCT scheme, and thereby propose the following hypothesis.

**Hypothesis 2** **(H2).** *Residents’ sense of social responsibility can positively influence their AAR towards the PCT scheme.*

The interpersonal behavior model concluded that individuals’ habits have significant effects on their behaviors [30]. The preference for comfort indicates a greater liking for living a comfortable life over making sacrifice for carbon emission reduction, which is an important factor of a resident’s carbon capability [31]. During the interviews of urban residents in China, many interviewees expressed that comfort and convenience are the most important factors in their daily lives, and they are not willing to make sacrifices in respect of these factors to improve their carbon capacities [31]. That is, the possibility of them adopting low-carbon behaviors is very low when residents have a strong preference for comfort. Therefore, it can be inferred that there are negative influences caused by residents’ preference for comfort on their AAR to the PCT scheme, and the hypothesis was made as follows.

**Hypothesis 3** **(H3).** *Residents’ preference for comfort can negatively influence their AAR towards the PCT scheme.*

The theory of responsible environmental behavior indicated that perceived knowledge of environmental action strategies was significantly influential in motivating individuals to adopt responsible environmental behaviors [23], and knowledge plays a fundamental role in creating environmental behaviors [22]. Some researchers also found that cognitions of climate change, low-carbon products, and ways for emissions reduction were fundamental psychological attributes of low-carbon behaviors, so people with high environmental awareness were usually willing to engage in low-carbon behaviors [32,33,34]. In this study, cognition of the behaviors for carbon emission reduction represents the psychological result of people perceiving, acquiring and intuiting the ways and behaviors for carbon emission reduction; cognition of PCT is defined as the psychological result of people perceiving, learning and reasoning PCT scheme. Based on the above analyses, residents are likely to approach the PCT scheme when they have high cognition of carbon emission reduction and the scheme. Hence, the following hypotheses were proposed.

**Hypothesis 4** **(H4).** *Residents’ cognition of the behaviors for carbon emission reduction can positively influence their AAR towards the PCT scheme.*

**Hypothesis 5** **(H5).** *Residents’ cognition of PCT can positively influence their AAR towards the PCT scheme.*

The theory of planned behavior shows that the behaviors and behavioral intentions of an individual are predicted by her/his subjective norms [19]. In general, subjective norms indicate the social pressure an individual feels about whether or not to take a particular action. For instance, Wei, Chen, and Long (2018) demonstrated that social norms, the social consumption culture, and social money could affect residents’ perceptions of the outcome of carbon capacity [31]. Based on the results of qualitative analyses, under the influence of the culture of consumerism, residents usually regard expensive goods and exclusive services as symbols of their status, which can easily result in the waste of energy; residents are probably low-carbon minded and willing to adopt low-carbon behaviors under the influence of the culture of environmentalism. Therefore, it can be assumed that the culture of consumerism will cause a negative effect on residents’ AAR towards the PCT scheme, while the culture of environmentalism will generate a positive effect on residents’ responses. Hence, hypotheses were proposed as follows.

**Hypothesis 6** **(H6).** *The culture of consumerism can negatively influence residents’ AAR towards the PCT scheme.*

**Hypothesis 7** **(H7).** *The culture of environmentalism can positively influence residents’ AAR towards the PCT scheme.*

The theory of planned behavior suggests that perceived behavioral controls are an important predictor of behavioral intentions or behaviors [19]. In this study, perceived behavioral control refers to the perceived ease or difficulty of participating in PCT. The results of in-depth interviews showed that residents perceived that their participation in PCT would result in the improvement of the environment, enhancement of their pleasure, an increase in their earnings (i.e., people get earnings by selling the remains of carbon permits, if they take low-carbon actions) as well as their cost of living. Hence, perceived behavioral control is assumed to mediate the relationships between psychological characteristics and responses, which can be explained as follows. Firstly, the theory of planned behavior pointed out that perceived behavioral control is the predictor of subsequent behaviors, and positive perceptions largely contribute to environmental actions [35,36,37]. Moreover, the perceived behavioral controls of residents were qualitatively verified to influence their behavioral willingness towards the PCT scheme. Secondly, individual factors were generally considered to affect their perceptions and experience of the performance of behaviors. Wei, Chen, and Long (2018) shed light on the influences on residents’ preferences for comfort and their ecological personality on their utility experience perceptions [31]. Thus, the perceived behavioral control of an individual is likely to be influenced by their psychological characteristics. Thirdly, the above analyses showed that the AAR of residents towards the PCT scheme could probably be influenced by their ecological values, sense of social responsibility, preference for comfort, cognition of the behaviors for carbon emission reduction, and cognition of personal carbon trading. Based on the above analyses, the following hypotheses could be put forward that the influence on residents’ responses of their psychological characteristics were likely to be mediated by their perceived behavioral controls.

**Hypothesis 8** **(H8).** *There are mediating effects of residents’ perceived behavioral controls on the relationships between their ecological values and AAR towards the PCT scheme.*

**Hypothesis 9** **(H9).** *There are mediating effects of residents’ perceived behavioral controls on the relationships between their sense of social responsibility and AAR towards the PCT scheme.*

**Hypothesis 10** **(H10).** *There are mediating effects of residents’ perceived behavioral controls on the relationships between their preference for comfort and AAR towards the PCT scheme.*

**Hypothesis 11** **(H11).** *There are mediating effects of residents’ perceived behavioral controls on the relationships between their cognition of the behaviors for carbon emission reduction and AAR towards the PCT scheme.*

**Hypothesis 12** **(H12).** *There are mediating effects of residents’ perceived behavioral controls on the relationships between their cognition of personal carbon trading and AAR towards the PCT scheme.*

### 2.3. Development Of Measurement Scales

Scale development is the prerequisite for questionnaire surveys, and it is closely related to the reliability and validity of the research results. The following steps were taken in order to develop measurement scales:

A. Determine the variables and propose hypotheses by means of a literature review, in-depth interviews, and expert consultation.
B. Make localized modifications of existing measurement scales, or design new scales based on the concepts of the related variables.
C. Conduct a preliminary investigation.
D. Test the reliability and validity of the initial scales.
E. Make amendments to the initial scales.
F. Establish formal measurement scales.

By means of the Wenjuanxing website (https://www.wjx.cn), the preliminary investigation was conducted from 9–28 October 2018. After deleting 68 invalid questionnaires, the final number of questionnaires was determined as 340. Furthermore, SPSS Statistics 22.0 was used to test the reliability of the initial scales. The Cronbach’s α coefficients for eight variables (i.e., AAR, ecological value, sense of social responsibility, preference for comfort, cognition of the behaviors for carbon emission reduction, cognition of PCT, perceived behavioral control, and culture of consumerism) were greater than 0.700, which suggests that these items had relatively high internal consistency. The Cronbach’s α coefficient for the culture of environmentalism was 0.685; thus, its reliability was considered to be acceptable. In order to improve the validity of the measurement scales, this study asked for advice from ten urban residents and ten experts in the field of energy economics and environmental management during the process of developing the scales. Altogether, the measurement scales were proven to have high reliability and validity.

The measurement items were as follows: (1) There were five items for the evaluation of the residents’ AAR to the PCT scheme; for example, “the personal carbon trading scheme can benefit future generations, and thus I am genuinely willing to accept it.” (2) The measurement items designed for the ecological value were “protect environment, prevent pollution, and live in harmony with nature.” (3) Four items were included for measuring the sense of social responsibility; for example, “I make great efforts for group interests.” (4) The preference for comfort was assessed by three items; for example, “I would not sacrifice my comfortable life for carbon emission reduction.” (5) There were four measurement items for cognition of the behaviors for carbon emission reduction such as “people can reduce carbon emissions by walking, riding a bicycle, taking a bus, and taking the subway” and “people can reduce carbon emissions by separating and recovering municipal solid waste.” (6) Three measurement items were designed for the cognition of personal carbon trading; for example, “the personal carbon trading scheme is to reduce the carbon emissions caused by households.” (7) The perceived behavioral control was measured by three items, for example, “I feel easy to participate in PCT scheme.” (8) There were three items developed for evaluating the culture of consumerism; for example, “it is very common that people consume goods and services in large quantities.” (9) The culture of environmentalism was assessed by three measurement items; for example, “many people attach great importance to energy conservation and emissions reduction.” In addition, the respondents were required to make choices based on their real thoughts and actual situations. The ecological value was rated on a five-point Likert scale: 5: very important, 4: slightly important, 3: neutral, 2: slightly unimportant, 1: very unimportant. The other variables were also rated on a five-point Likert scale: 5: strongly agree, 4: slightly agree, 3: undecided, 2: slightly disagree, 1: strongly disagree.

### 2.4. Description of Investigation Process

Urban residents in eastern China were selected as respondents, because China is the largest contributor to global carbon emissions and the urban area concentrates most of the emission load [38]. High income residents produce a considerable amount of carbon emissions due to their large energy demands, and they can usually afford to buy energy-saving but expensive appliances; thus, they play a key role in the abatement of carbon emissions and the implementation of the PCT scheme. The PCT is a forward-looking scheme that requires participants to have a high sense of social responsibility, general awareness of energy conservation and emissions reduction, and a strong ability to learn. Furthermore, the China Statistical Yearbook 2018 showed that the per capita gross domestic product in Beijing, Shanghai, Tianjin, Jiangsu, Zhejiang, Fujian, and Guangdong was the highest in the country. Therefore, the urban residents in the eastern region, which includes seven provinces (i.e., Fujian, Guangdong, Hainan, Hebei, Jiangsu, Shandong, and Zhejiang) and three municipalities directly under the central government (i.e., Beijing, Shanghai, and Tianjin) were selected as the respondents for this study.

Formal online and offline investigations were conducted from 10 November 2018 to 10 January 2019. In order to ensure the validity of the data, several measures were adopted during the data collection process. Firstly, an agreement was made between the researchers and the Wenjuanxing online survey website to avoid information leakage, and every respondent was fully informed. Secondly, an announcement in respect of data security appeared in the opening part of every questionnaire; that is, that the information collected would only be used for scientific purposes. Thirdly, monetary payoffs were the main incentive mechanism to arouse respondents’ enthusiasm. Specifically, every online respondent was paid through the survey website, and the amount was randomly assigned in the range of 1–2 Chinese Yuan, which served as a stimulus to enhance the respondents’ motivation and to strengthen their patience; every offline respondent was offered 2 Chinese Yuan (i.e., approximately 0.3 US Dollar) as a reward for participation. It is noteworthy that the offline survey was conducted in order to include old and low-education-level residents. For some elder respondents, this study used the one-to-one interview, in which interviewers fill out questionnaires according to the answers given by respondents. In addition, a filtering process was employed to remove invalid data. If one answer was missing or eight consecutive answers were the same, the questionnaire was recognized as an invalid one [39]. Finally, 403 questionnaires were deleted to ensure the quality of the data. To sum up, the total number of valid questionnaires was 1892, and the effective recovery rate reached 82.44%.

## 3. Results

### 3.1. Descriptive Statistics

The descriptive statistics of the variables are shown in Table 2 and Table 3. The residents holding approach responses towards the PCT scheme accounted for 74.10% of the total, and the mean value of the AAR reached 3.79, which implied that, in general, the residents had an approach response to the PCT scheme. The mean of the ecological value was 4.43, which signified the great emphasis the residents placed on the ecological environment. It was unexpected that the culture of environmentalism and consumerism prevailed simultaneously in eastern China; both mean values were greater than 3.00. The standard deviation of preference for comfort was quite large, so it was very likely that there would be a big difference in the preference for a comfortable life among individuals. The great mean value of the cognition of the behaviors for carbon emission reduction indicated that the residents generally knew how to save energy and reduce carbon emissions.

### 3.2. Demographic Distribution of Respondents

Table 4 shows the demographic distribution of the 1892 respondents. The male and female respondents accounted for 52.33% and 47.67%, respectively. The sex ratio is nearly consistent with reality based on the “China Statistical Yearbook 2020” complied by the National Bureau of Statistics of China. In the age categories, respondents aged 21–30 accounted for 45.77% of the total, which was the highest proportion. Overall, the respondents are concentrated at the middle age intervals, with fewer at both ends. Respondents with a bachelor’s degree made up the largest proportion of the total, which is consistent with the demographic statistics of three cities—Shenzhen, Nanjing, and Fuzhou. The reason behind this selection of cities is the representativeness of areas with different development levels. Among the annual individual income (i.e., the income earned by the respondent herself/himself) categories, the distribution is substantially in line with the economic facts of eastern Chinese cities as revealed by an authoritative report [40].

**Figure 1:**
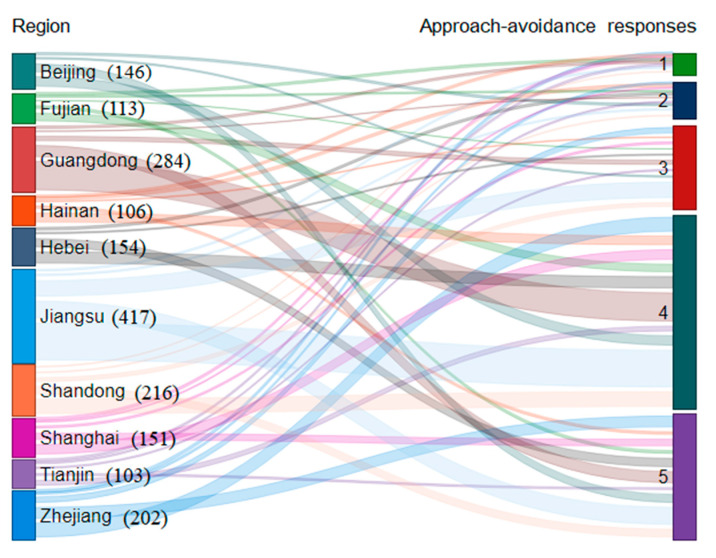
The regional distribution of the samples and the regional contribution to each level of approach–avoidance responses (AAR).

The regional distribution of samples and the regional contribution to each level of AAR are two important points for the sample’s characterization. In order to present these data in an efficient way, we adopted the Sankey diagram in Figure 1. The width of the curve is proportional to the regional contribution to each level, hence the most important contributions are readily located by comparing the width of curves. The left side of this diagram showed the regional distribution of the respondents: 146 respondents in Beijing, 113 respondents in Fujian, 284 respondents in Guangdong, 106 respondents in Hainan, 154 respondents in Hebei, 417 respondents in Jiangsu, 216 respondents in Shandong, 151 respondents in Shanghai, 103 respondents in Tianjin, and 202 respondents in Zhejiang. The right side of Figure 1 demonstrated that a large proportion of the respondents in eastern China were willing to accept the PCT scheme, because node “4” showed the maximum width, closely followed by node “5”. Through tracing the curves of node “5”, it could be found that respondents from Jiangsu province hold the most positive response to PCT scheme, and the second most positive is Zhejiang province.

### 3.3. Regression Analysis

Multiple linear regression (software: SPSS Statistics 22.0) was adopted to examine the relationships between residents’ psychological characteristics, subjective norms and their AAR towards the PCT scheme, and the results are shown in Table 5. The model was significant at the 0.01 level, the adjusted R^2^ reached 0.469; thus, the model fitted the data well. Furthermore, the residents’ AAR were positively correlated with the ecological value, sense of social responsibility, cognition of the behaviors for carbon emission reduction, and cognition of PCT at the 0.01 level of significance; thus, the results supported hypotheses H1, 2, 4, and 5. There was a significantly negative relationship between preference for comfort and AAR, which supported hypothesis H3. These results indicated that the residents increased their positive response towards the PCT scheme as they enhanced their ecological value, sense of social responsibility, cognition of the behaviors for carbon emission reduction, and cognition of PCT, and they decreased their preference for comfort. Thus, there were significant influences caused by the residents’ psychological characteristics on their AAR towards the PCT scheme. In addition, residents’ AAR to PCT scheme were negatively impacted by the culture of consumerism, while they were positively influenced by the culture of environmentalism. Therefore, hypotheses H6 and 7 were supported, and the culture of environmentalism was demonstrated to be an effective push for residents towards the PCT scheme.

To examine the mediating effects of perceived behavioral controls, the following three steps were adopted. The first was to examine the coefficients ($c_{1}$) of the psychological factors in the regression model of psychological characteristics and AAR. There were no mediating effects if $c_{1}$ was not significant. The second step was to measure the coefficients (a) of the psychological factors in the regression model of psychological characteristics and perceived behavioral controls. The third was to estimate the coefficients of the psychological factors ($c_{2}$) and perceived behavioral controls (b) in the regression model of psychological characteristics, perceived behavioral controls, and AAR. Table 6 reports the mediating effect test results of perceived behavioral controls. It shows that the a was not significant and the b was significant in the path EV→PBC→AAR. Thus, this study applied the Z-test to conduct Sobel test: $Z=\hat{a}\hat{b}/S_{ab}$. Specifically, $S_{ab}=\sqrt{{\hat{a}}^{2}S_{b}{}^{2}+{\hat{b}}^{2}S_{b}{}^{2}}$; $\hat{a}$ and $\hat{b}$ indicated the estimation of a and b, respectively; $S_{a}$ and $S_{b}$ denoted the standard error, respectively [41]. The calculated z ($-0.031\times 0.387/\sqrt{{\left(-0.031\right)}^{2}\times 0.021^{2}+0.387^{2}\times 0.033^{2}}=-0.9382$) did not exceed −0.90 using the above formula, which implies that the null hypothesis could not be rejected with a 0.95 confidence interval [42]. Hence, hypothesis H8 was supported, and the perceived behavioral controls played a mediating role in the relationship between the ecological value and the AAR. In addition, all the coefficients were significant at the 0.01 level in the other paths, which indicated that the relationships between the sense of social responsibility, preference for comfort, cognition of the behaviors for carbon emission reduction, and cognition of PCT and AAR could be mediated by the perceived behavioral controls; thus, hypotheses H9, 10, 11, and 12 were supported. Moreover, this study measured the proportion of the mediating effect to the total effect. For example, in the path SSR→PBC→AAR, the direct effect of the sense of social responsibility could be expressed by 0.107 ($c_{2}=0.107$), the mediating effect was calculated as 0.089 (ab=0.229×0.387=0.089), the total effect was 0.196 ($c_{1}=0.196$), and the proportion of the mediating effect could be calculated as 45.41% (0.089/0.196×100%=45.41%). Therefore, the above results supported hypotheses, and there was a mediating effect caused by the residents’ perceived behavioral controls on the relationships between the psychological characteristics and the AAR to the PCT scheme.

## 4. Discussion

The PCT scheme is regarded as an innovative and forward-looking tool for downstream carbon emission reduction, but a major obstacle to its implementation is public acceptability. Taking a sample of urban residents in eastern China as respondents, this study found that 74.10% of them made approach responses towards the scheme, 17.65% showed neutral attitudes, and only 8.25% of respondents reported avoidance responses. The evidence elsewhere has shown that attitudes towards the PCT scheme have varied considerably from country to country. For example, 13% of the college students and residents in Cambridge, United Kingdom expressed support for a PCT scheme [12]. In Tianjin, China, 74.92% of the residents agreed to participate in the PCT [14]. Tianjin is located in the eastern part of China. Thus, it can be inferred that the vast majority of urban residents in eastern China were willing to accept the PCT scheme. Additionally, this study found that many residents took a wait-and-see attitude due to their lack of knowledge about the PCT scheme. Von (2008) also indicated that people with a neutral attitude to the PCT scheme accounted for 42% of the total [12]. Therefore, a lot of advance publicity about the PCT scheme should be given to improve public acceptability, which would thereby remove a serious obstacle to implementing the PCT scheme.

It is demonstrated that the residents can be driven into approaching the PCT scheme by the culture of environmentalism. This provides a theoretical basis for the subsequent policy interventions. The target of carbon emission reduction cannot be reached solely by the consciousness of residents [36,43]. External interventions are necessary for the enhancement of residents’ positive responses to the PCT scheme. Researchers also found that social learning, social comparison, and social identity play positive roles in low-carbon lifestyles [43]. In addition, Guangdong province is piloting the Carbon Generalized System of Preferences, but the current result is unsatisfactory. A possible reason is that the guidance for residents’ low-carbon behaviors is still from the public perspective rather than the target-oriented multi-means intervention. In view of the current situation, future research focusing on intervention policies should take the effects of environmentalism and consumerism into account, since the willingness to trade sustainably originates from voluntary participation in the PCT scheme.

## 5. Conclusions and Suggestions

### 5.1. Conclusions

The responses from the sampled urban residents in respect of the PCT scheme were as follows: 74.10% made an approach response, 17.65% declared neutrality, and only 8.25% reported an avoidance response. The determinants of residents’ AAR towards the PCT scheme mainly included psychological characteristics, perceived behavioral controls and subjective norms. Firstly, the AAR of the residents towards the PCT scheme were significantly influenced by the psychological characteristics. Specifically, their responses were positively affected by their ecological values, sense of social responsibility, cognition of the behaviors for carbon emission reduction, and cognition of PCT, whereas they were negatively influenced by their preference for comfort. Secondly, residents’ subjective norms significantly influenced their AAR towards the PCT scheme. The culture of consumerism posed a negative impact on residents’ responses, while the culture of environmentalism had a positive effect on their responses. Thirdly, there were mediating effects caused by the residents’ perceived behavioral controls on the relationships between their psychological characteristics and AAR towards the scheme.

### 5.2. Policy Suggestions

Based on the above results, the following primary and pivotal suggestions are proposed for appropriately nudging urban residents towards approaching the PCT scheme.

Primary suggestions. The results showed that the residents’ responses to the PCT scheme were primarily influenced by their psychological characteristics and subjective norms; thus, this study proposed primary policy suggestions. Ecological value is an important motivation for taking environmental actions. It is difficult to change the values of a person once they are formed, but they can be cultivated from childhood. Therefore, this study suggested that the government should attach great importance to the initial shaping of ecological values. The sense of social responsibility is a driving force for residents to accept the PCT scheme. The results of the in-depth interviews indicated that some residents laid too much stress on self-responsibility and had a weak sense of social responsibility. Hence, much attention should be devoted to the cultivation and enhancement of residents’ sense of social responsibility. The preference for comfort is a major obstacle to the implementation of the PCT scheme. Residents should be guided to rationally pursue the comforts of life in order to reduce the carbon emissions caused by reckless consumption and to improve the public acceptability of the scheme. Additionally, the results of the in-depth interviews reflected that some people barely knew how to reduce carbon emissions in their daily life, and they didn’t even have much understanding of the PCT scheme. Therefore, specific actions need to be taken to improve residents’ cognition of the ways to reduce carbon emissions and how the scheme facilitates this process. The culture of environmentalism indicates the social forms of a society in which people are encouraged to adopt low-carbon and pro-environmental behaviors, and it was proved to be an important factor for approaching the PCT scheme. The government should give more attention to the environmental and nature-related aspects of green ideology, which is beneficial for creating a low-carbon society.

Pivotal suggestions. The perceived behavioral controls were proved to be pivotal in the AAR of residents towards the PCT scheme. Based on the results of in-depth interviews and regression analysis, pivotal suggestions should be proposed to provide guidance for urban residents in approaching the PCT scheme. For example, a pilot program could be started to make residents perceive that participation in the PCT can deliver economic benefits, improve their living environment, and give spiritual satisfaction.

## Figures and Tables

**Table 1:** Definitions of the influencing factors.

Influencing Factors	Definition
Ecological value	The perceived importance of improving environmental quality.
Sense of social responsibility	The awareness of having an obligation to make contributions to the society.
Preference for comfort	The greater liking for living a comfortable life over making sacrifice for carbon emission reduction.
Cognition of the behaviors for carbon emission reduction	The psychological result of people perceiving, acquiring and intuiting the ways and behaviors for carbon emission reduction.
Cognition of PCT	The psychological result of people perceiving, learning and reasoning PCT scheme.
Perceived behavioral control	The perceived ease or difficulty of participating in PCT and it is assumed to reflect past experience and anticipated impediments.
Culture of consumerism	The social forms of a society that people are encouraged to acquire goods and services in ever-increasing amounts.
Culture of environmentalism	The social forms of a society that people are encouraged to adopt low-carbon and pro-environmental behaviors.

**Table 2:** Descriptive statistics of the approach–avoidance responses to personal carbon trading.

Variable	Mean	Standard Deviation	Proportion		
			Avoidance (Denoted by “1” and “2”)	Neutrality (Denoted by “3”)	Approach (Denoted by “4” and “5”)
AAR ^1^	3.79	0.90	8.25%	17.65%	74.10%

^1^ AAR: approach–avoidance responses.

**Table 3:** Descriptive statistics of other variables.

Variable	EV ^1^	SSR ^2^	PC ^3^	CBCER ^4^	CPCT ^5^	PBC ^6^	CC ^7^	CE ^8^
Mean	4.43	3.83	2.87	4.19	3.84	3.61	3.68	3.59
Standard deviation	0.72	0.85	1.03	0.80	0.85	0.80	0.92	0.86

^1^ EV: ecological value; ^2^ SSR: sense of social responsibility; ^3^ PC: preference for comfort; ^4^ CBCER: cognition of the behaviors for carbon emission reduction; ^5^ CPCT: cognition of personal carbon trading; ^6^ PBC: perceived behavioral control; ^7^ CC: culture of consumerism; ^8^ CE: culture of environmentalism.

**Table 4:** The demographic distribution of the 1892 respondents.

Variable	Classification	Number	Proportion
Gender	Male	990	52.33%
	Female	902	47.67%
Age	18–20-years-old	75	3.97%
	21–30-years-old	866	45.77%
	31–40-years-old	551	29.12%
	41–50-years-old	250	13.21%
	Older than 50-years-old	150	7.93%
Education level	Middle school education and below	81	4.28%
	High school education	117	6.18%
	College degree	161	8.51%
	Bachelor’s degree	711	37.58%
	Master’s degree	644	34.04%
	Doctorate degree	178	9.41%
Annual individual income	Less than 30,000 Chinese Yuan	429	22.68%
	30,000–50,000 Chinese Yuan	180	9.51%
	50,000–80,000 Chinese Yuan	222	11.73%
	80,000–100,000 Chinese Yuan	310	16.39%
	100,000–150,000 Chinese Yuan	348	18.39%
	150,000–200,000 Chinese Yuan	152	8.03%
	200,000–300,000 Chinese Yuan	129	6.82%
	300,000–500,000 Chinese Yuan	77	4.07%
	500,000–1,000,000 Chinese Yuan	45	2.38%

Note: Chinese Yuan is approximately equal to 0.15 US Dollar.

**Table 5:** The regression results of psychological factors on approach–avoidance responses.

Variable	Unstandardized Coefficient	Standard Error	T	Sig.
Constant	0.907 ***	0.156	5.812	0.000
EV ^1^	0.138 ***	0.031	4.432	0.000
SSR ^2^	0.196 ***	0.030	6.432	0.000
PC ^3^	−0.106 ***	0.020	−5.405	0.000
CBCER ^4^	0.176 ***	0.029	6.087	0.000
CPCT ^5^	0.284 ***	0.025	11.480	0.000
CC ^6^	−0.128	0.017	−7.383	0.000
CE ^7^	0.068	0.025	2.740	0.006
Adjusted R^2^	0.469			
F	239.244 ***			

^1^ EV: ecological value; ^2^ SSR: sense of social responsibility; ^3^ PC: preference for comfort; ^4^ CBCER: cognition of the behaviors for carbon emission reduction; ^5^ CPCT: cognition of personal carbon trading; ^6^ CC: culture of consumerism; ^7^ CE: culture of environmentalism; *** represent the level of significance at 0.01.

**Table 6:** Mediating effect test results of perceived behavioral controls.

Path	$c_{1}$	a	b	$c_{2}$	Is there a Mediating Effect?	Proportion of Mediating Effect
EV ^1^→PBC ^6^→AAR ^7^	0.138 ***	−0.031	0.387 ***	0.150 ***	Yes	8.69%
SSR ^2^→PBC→AAR	0.196 ***	0.229 ***	0.387 ***	0.107 ***	Yes	45.41%
PC ^3^→PBC→AAR	−0.106 ***	−0.138 ***	0.387 ***	−0.053 ***	Yes	50.38%
CBCER ^4^→PBC→AAR	0.176 ***	0.174 ***	0.387 ***	0.109 ***	Yes	38.26%
CPCT ^5^→PBC→AAR	0.284 ***	0.423 ***	0.387 ***	0.121 ***	Yes	57.64%

^1^ EV: ecological value; ^2^ SSR: sense of social responsibility; ^3^ PC: preference for comfort; ^4^ CBCER: cognition of the behaviors for carbon emission reduction; ^5^ CPCT: cognition of personal carbon trading; ^6^ PBC: perceived behavioral control; ^7^ AAR: approach–avoidance responses; *** represent the level of significance at 0.01.

## Data Availability

The data presented in this study are available on request from the first author.
